# Factors related to the non-adherence of medication and nonpharmacological recommendations in high blood pressure patients

**DOI:** 10.15171/jcvtr.2019.05

**Published:** 2019-02-28

**Authors:** Farzane Etebari, Mohammad Zakaria Pezeshki, Sanam Fakour

**Affiliations:** ^1^Department of Community Medicine, Tabriz University of Medical Sciences, Tabriz, Iran; ^2^Medicine Faculty, Tabriz University of Medical Sciences, Tabriz, Iran

**Keywords:** Adherence, Diet, Education, Hypertension, Income, Socioeconomic Factors

## Abstract

***Introduction:*** Many studies have been conducted on non-adherence with the antihypertensive treatment regime in various countries, considering the burden of cardiovascular disease (CVD) on the public health system, it is essential to carry out studies in this regard.

***Methods:*** Patients with hypertension evaluated at the family medicine clinic of Tabriz University of Medical Sciences were enrolled using simple sampling. Data gathering tool was a questionnaire consisting of three sections including the Hill-Bone compliance questionnaire, the disease characteristics, and patients’ socioeconomic.

***Results:*** Of 254 patients with hypertension, gender, income satisfaction, the occupation and the level of education did not correlate with the acceptance of the treatment. However, the number of antihypertensive medications had a significant effect on adherence with dietary orders and appointment keeping (*P*<0.01 and *P*=0.01, respectively). The number of antihypertensive drugs could statistically significantly predict overall score obtained from the questionnaire, F (1, 251) = 22.29, *P*<0.018.

***Conclusion:*** Factors related to the history of the disease and socioeconomic status had no effect patients adherence with treatment; however, the number of the prescribed antihypertensive drugs is in association with higher overall scores obtained through the Hill-Bone questionnaire.

## Introduction

Cardiovascular diseases (CVDs) inflict high burden of the direct costs on the medical system, as well as the accounting for increased rate of the morbidity and mortality incidence annually.^1^ On this basis, it has been established that antihypertensive drugs lead to significant improvement of the coronary heart diseases (CHDs), myocardial infarctions (MIs), and congestive heart failures (CHF).^2,3^ Thus, management of the high blood pressure is of essential importance to reduce the risk of the correlated complications the minimum. However, despite several strategies for the prevention and treatment of hypertension (HTN) that has taken into consideration, poor adherence to long-term prescription have reported resulting in disappointing rates for the blood pressure control.^4^ Whereas, according to the literature, only 20% of the patients who have been prescribed to receive antihypertensive drugs, have sufficient adherence to benefit the treatment outcomes, with a high rate of decline in adherence and discontinuation of the treatment during first six months.^5^ Considering lowering blood pressure role in the reduction of the CVD, failure to control HTN might result in several life-threatening complications such as stroke, nephropathies and, MI.^6,7^

However, the self-management of the HTN provides a health-care approach in which the patient plays a pivotal role in promoting health, complications prevention, and successful disease control concerning the acceptable adherence with the antihypertensive therapy. Successful maintaining of self-management includes the demand for change in behavior or lifestyle.^8^ Lack of adherence with the antihypertensive therapy has been introduced to play the primary role in poorly controlled BP development and incidence of the subsequent complications. On this basis, unfortunately, most investigations have been limited to asking for drug consumption adherence without concerning the underlying factors that might have influence, not only on therapy adherence but also on consulting the physician and adhering to the dietary recommendations.

Whereas, one of the behaviors that predict successful treatment or reduces the severity of the illness is to adhere to the therapeutic recommendations provided by the physicians to the patient.^9^ Although a significant amount of expenses and time is spent on disease diagnosis, many patients neglect the recommended diet regimen or further suggestions.^10^ Many individuals experience several problems in adhering to long-term treatment. Reports suggest that poor adherence to treatment can increase the cost of the treatment for hypertensive by 15%-20%, leading to frequent hospitalization, referring to the emergency rooms and admission to the intensive care unit.^11^ So that, in the literature, some studies have suggested a strong correlation between proper adherence with the therapy and improvement of the HTN control and long-term outcomes.

Many studies have been carried out on antihypertensive treatment regardless of the dietary recommendations in the developed countries, and few studies considered alcohol consumption effect without concerning the countries with restrictions for alcoholic beverages.^12,13^ Additionally, very few studies have been conducted on the association of the adherence to the dietary regimens, antihypertensive medication and physician consulting, especially in the low- and middle-income countries. Furthermore, due to the significant burden of CVD on the public health system, the demand for studies in this area is quite perceptible. Therefore, this study aimed to investigate the factors associated with the nonadherence of pharmaceutical and non-pharmaceutical recommendations in patients with HTN for the first time in Tabriz family medicine clinic.

## Materials and Methods

### Participants

An observational study carried out in Tabriz University of Medical Sciences family medicine clinics. In this study, the sample size determined via the correlation sample size formula N: [ (*Z*_α_ + Z_β_)/C]^2^+3. Whereas, considering N as study population, *Z*_α_ as the standard normal deviate for the type I error rate (0.050), Z_β_ as standard normal deviate for the type II error (0.200), and C calculated by expected correlation efficiency rate (r) of (according to the study conducted in outpatient individuals in the United States, the correlation coefficiency between marital status and medication adherence among hypertensives was 0.19),^14^ the study sample size calculated to include 215 hypertensive patients, while considering 10% of probability for loss of data, 236 hypertensive patients estimated to participate in the study. Patients enrolled via simple random sampling among hypertensive patients.

### Inclusion and exclusion criteria

Patients with HTN consulting to the family medicine clinic aged 30 years consuming anti-hypertensive drug therapy. Patients who did not have the tendency to participate in the study or had a history of psychologic and cognitive impairments excluded.

### Study design and materials

The data collection tool in this study was a questionnaire consisting of three sections, as follows: the first part of which was the Hill-Bone compliance to high blood pressure therapy scale that was designed by Kim et al in 2000 including 14 items.^15^ The scale mentioned above was covering three areas including adherence on drug regimens, diet regimen, and follow up on medical appointments, as follows: follow up of drug recommendations (8 questions), follow up the diet (three questions) and keep track of the promise of meeting with the doctor (three questions). Each question had five options in reverse, in which the score of 5 considered as “I never forget,” and the score of 1 determined as “I always forget.” The overall minimum and maximum scores of the questionnaire were 14 and 70, respectively.^16^ The Hill-Bone compliance questionnaire was translated from English to Persian via the standard backward-forward method by Gholami and colleagues. The reliability of the tool was determined according to the study done by Zabihi et al and the Cronbach’s alpha value was reported to be 0.71 for the Hill-Bone questionnaire.^17,18^ The validity of this questionnaire was confirmed by content validity method by 12 faculty members of the Nursing and Midwifery school of the Shahid Beheshti, and the reliability of the questionnaire was confirmed by calculating the Cronbach’s alpha coefficient of 0.8.^19^

The second section of the data collection tool included the characteristics of the disease concerning the duration of the diagnosis, the family history of HTN in the first-degree relatives, the intervals of referral to the doctor, the number of blood pressure drugs and suffering from the complications followed by high blood pressure. Accordingly, in the third section of the questionnaire, items such as demographic and socioeconomic factors related to the disease were collected including age, sex, marital status, occupational status, educational level, education level of the spouse, benefiting insurance, household-level, and satisfaction from family income was recorded. With due attention to the study by Howe et al., individual, household-level, and education level were the main factors in the assessment of the socioeconomic position of the participants in low- and middle- income countries, that explains the variables inserted in the questionnaire.^20^

### Statistical analysis

The chi-square test used for comparing two qualitative variables in each time, and student *t* test for comparing quantitative variables between groups. The level of significance was set at 0.05, and all results were expressed by frequency (percent) for qualitative variables and mean ± SE for quantitative variables.

Linear regression was run to understand the effect of age, gender, the length of the disease, and the number of prescribed antihypertensive on the overall score of the Hill-Bone questionnaire score. To assess linearity, a scatterplot of the overall score against abovementioned variables with superimposed regression line was plotted. Visual inspection of these two plots indicated a linear relationship between the variables. There were homoscedasticity and normality of the residuals. There was no outlier during the analysis.

Multiple regression was run to predict the overall score of the Hill-Bone questionnaire from gender, age, marital status, employment status, patients’ and spouse’s education level, type of medical insurance, income satisfaction, familial history of HTN, number of antihypertensive drugs, blood pressure self-measurement, disease length, and comorbidities. There was linearity as assessed by partial regression plots and a plot of studentized residuals against the predicted values. There was the independence of residuals, as assessed by a Durbin-Watson statistic of 2.009. There was homoscedasticity, as assessed by visual inspection of a plot of studentized residuals versus unstandardized predicted values. There was no evidence of multicollinearity, as assessed by tolerance values greater than 0.1. There were no studentized deleted residuals greater than ±3 standard deviations, no leverage values greater than 0.2, and values for Cook’s distance above 1. The assumption of normality was met, as assessed by a Q-Q Plot.

## Results

In this study, 255 patients with HTN referred to the Family Medicine Clinic of the Tabriz University of Medical Sciences were evaluated for inclusion in the study. One patient was excluded from the study due to incomplete data. Statistical analyzes were performed on 254 other patients. The mean age of the patients participating in the study was 58.16 ± 10.54. The demographic and socioeconomic data and level of education of all patients and 206 one of the patients’ spouses are shown in Tables 1 and 2 respectively. Regarding economic status and financial rehabilitation of the patients, household-level and income satisfaction were asked and the outcomes summarized in Table 1.

**Table 1:** Patients’ demographic and socioeconomic position data with Hill-Bone compliance questionnaire scores compared using independent *t* test

		**No. (%)**	**Hill-Bone compliance questionnaire**							
			**Overall**	***P***	**Dietary**	***P***	**Appointment**	***P***	**Medication**	***P***
Gender	Female	110 (43.3)	35.51±9.3	0.49	7.79±2.18	0.45	8.44±2.75	0.65	19.28±5.65	0.72
	Male	144 (56.7)	36.31±9.1		7.88±1.74		8.59±2.64		19.84±6.02	
Income satisfaction	No	207 (81.5)	36.38±8.85	0.31	7.91±1.9	0.27	8.57±2.57	0.57	19.79±5.78	0.25
	Yes	47 (18.5)	34.6±10.49		7.55±2.09		8.3±3.16		18.74±6.2	
Hypertension familial history	No	83 (32.7)	37.8±9.26	0.2	8.11±1.97	0.15	8.57±2.54	0.86	20.41±5.99	0.16
	Yes	160 (63)	35.53±8.89		7.74±1.89		8.51±2.71		19.28±5.67	
Hypertension complications	No	87 (34.3)	34.95±9.43	0.2	7.71±1.88	0.26	8.21±2.47	0.17	19.03±6.2	0.44
	Yes	167 (65.7)	36.49±9.03		7.91±1.97		8.69±2.78		19.89±5.68	
Self-monitoring	No	85 (33.5)	35.72±9.58	0.76	7.91±2.1	0.63	8.46±2.8	0.78	19.35±6.09	0.71
	Yes	169 (66.5)	36.09±9		7.81±1.86		8.56±2.63		19.72±5.76	
Household level	Land- owner	186 (73.2)	36.35±9.43	0.33	7.84±1.98	0.12	8.52±2.76	0.92	19.89±6.03	0.98
	Tenant	68 (26.8)	34.99±8.6		7.84±1.87		8.55±2.55		18.6±5.35	

Of the patients participating in the study, 243 patients were aware of the familial history of HTN in first-degree relatives, whereas 160 (65.8%) patients had a positive history. The history HTN complications including, CVD and other chronic diseases were positive 167 (65.7%) patients. There was a wide range considering the length of HTN suffering in patients, which the minimum and maximum disease duration were six months and 30 years, respectively, however, the mean duration of HTN incidence in patients was 5.85 ± 8.23 ​​years.

The number of antihypertensive drugs consumed by patients is reported in table 2. Additionally, 169 (66.5%) patients had blood pressure monitoring. Regarding time interval between patients’ referral to the physician for follow-up sessions and control of blood pressure, 16 (6.2%) patients reported consulting only during lack of proper control of HTN. We also considered the patients who had a regular time interval of three or six months or one or two years. The mean consulting intervals of patients was 6.61 ± 8.05 months.

**Table 2:** Patients’ educational status, health insurance type and etc. with Hill-Bone compliance questionnaire scores compared via ANOVA

‏	‏	**No. (%)**	**Hill-Bone compliance questionnaire**							
			**Overall**	***P***	**Dietary**	***P***	**Appointment**	***P***	**Medication**	***P***
Marital status	Single	11 (4.3)	38±11.31	0.07	10±1.41	0.64	8.5±2.12	0.06	19.5±7.78	0.08
	Married	195 (76.8)	33.18±8.81		7.18±2.04		7.27±2.41		18.73±5.06	
	Widow	41 (16.1)	36.75±8.95		7.89±1.94		8.73±2.6		20.13±5.81	
	Divorced	5 (2)	32.93±9.7		7.68±1.74		7.8±3.02		17.44±6.02	
Occupation	Employed	44 (17.3)	37.11±8.47	0.24	8.19±1.88	0.24	9.22±2.97	0.3	19.69±5.24	0.26
	Self-employed	36 (14.2)	36.09±9.31		7.88±1.71		8.46±2.72		19.76±6.21	
	Housewife	123 (48.4)	37.14±9.47		7.92±2.28		8.65±2.63		20.57±5.87	
	Retired	51 (20.1)	35.6±11.39		7.8±3.27		9.2±3.03		18.6±5.59	
Patient's educational level	Non-educated	141 (55.5)	36.73±9.28	0.11	8±1.98	0.55	8.71±2.62	<0.01	20.02±5.98	0.44
	Elementary	22 (8.7)	31.82±9.35		7.27±1.78		6.59±2.65		17.95±6.41	
	Middle-school	21 (8.3)	38.29±8.71		8.14±1.53		10±2.59		20.14±5.74	
	High school diploma	49 (19.3)	34.84±9.12		7.53±2.02		8.22±2.72		19.08±5.73	
	College diploma	4 (1.6)	36.5±5.45		7.75±0.96		10±0.82		18.75±5.25	
	Bachelor	16 (6.7)	36.92±8.63		8.08±2.4		8.77±2.01		20.08±4.92	
	Master	1 (0.4)	21		7		4		10	
Patient's spouse educational level	Non-educated	99 (39)	33.36±9.85	0.58	7.54±2.03	0.71	7.85±2.79	0.67	17.96±5.99	0.42
	Elementary	7 (2.8)	37.56±9.22		7.98±2		8.87±2.73		20.71±5.79	
	Middle-school	36 (14.2)	38.14±10.73		8.71±1.98		8.29±3.4		21.14±6.87	
	High school diploma	52 (20.5)	36.11±8.07		7.69±1.82		9.03±2.55		19.39±5.1	
	College diploma	5 (2)	35.38±8.72		7.96±1.85		8.19±2.56		19.23±5.99	
	Bachelor	7 (2.8)	36±12.98		7.4±2.88		8.4±2.41		20.2±7.95	
Health insurance	Work insurance	75 (29.5)	36.36±8.91	0.23	8.14±1.46	0.57	8.52±2.61	0.02	19.99±5.48	0.33
	Governemental	7 (2.8)	35.14±7.65		7.78±1.97		7.43±2.37		19.57±4.47	
	Self-issued	153 (60.2)	37.89±7.77		8.67±1.58		8.64±2.68		19.54±6.02	
	Military	9 (3.5)	20.5±0.71		7.4±2.88		9.67±2.78		19.56±4.67	
	Others	10 (4)	36±12.98		7.29±0.76		8.4±2.41		20.2±7.95	
Number of drugs	One	126 (49.6)	34	0.03	10	0.18	8	<0.01	16	0.01
	Two	110 (43.3)	35.01±9.22		7.68±1.91		8.34±2.63		18.98±5.99	
	Three	17 (6.7)	37.66±8.31		8.17±1.89		9±2.54		20.49±5.33	

Abbreviations: ANOVA, analysis of variance. *P <0.05.

As previously mentioned, the Hill-Bone compliance questionnaire was used to assess the adherence of the medication and non-pharmacological treatment. The average overall patient score was 35.96 ± 9.17 according to the responses given, of which 14 and 51 were the minimum and maximum scores, respectively. Also, the scores for each of the three discipline of the questionnaire were calculated based on the patient’s responses, which the minimum, maximum and average values ​​are reported in Table 3.

**Table 3:** Hill-Bone compliance questionnaire overall and divided scores mean ± SD values

** **	**Score**	**Minimum**	**Maximum**	**Mean ± SD**
Hill-Bone compliance questionnaire	Overall	14	51	35.96±9.18
	Dietary	3	14	7.84±1.94
	Medication	8	31	19.6±5.86
	Appointment	3	13	8.52±2.68

Abbreviations: SD, standard deviation.

To evaluate the patients’ adherence to non-pharmacological drug recommendations in patients with high blood pressure, we compared the overall scores and the scores of the three fields of the Hill-Bone questionnaire based on demographic, socioeconomic and medical history data. The results of the statistical analysis are summarized in Tables 1 and 2. No significant difference observed while comparing the scores of the patients according to the gender, income satisfaction, residency status, familial history of HTN, the presence of HTN complications and self-monitoring of the blood pressure. Also, patients’ scores showed no significant difference concerning patients’ employment and occupational status and the educational level of the patient and his or her spouse, (Tables 1 and 2).

Similarly, the results of comparing patient scores based on the type of medical insurance showed no significant difference between the overall scores, as well as dietary recommendation and drug consumption adherence scores obtained from the questionnaire. However, the type of patient’s health insurance led to a significant difference regarding the score of the referral to the physician (*P* = 0.02).

On the other hand, a comparison of the scores based on the number of administered antihypertensive drugs showed a significant difference between the overall scores obtained from the questionnaire, adherence to dietary orders and the scores of the regular follow up sessions and consulting the physician (*P* < 0.01, *P* = 0.01, *P* = 0.03, respectively). Thus, patients divided to groups based on the number of drugs, and the analysis results showed that patients who administered less than two antihypertensive drugs acquired higher scores concerning the adherence with dietary guidelines, as well as adherence to regular referral to the physician (*P* = 0.02, *P* = 0.02, sequentially).

By enrolling significant variables of the primary analysis, we tried to investigate the effect of each factor on the patients’ overall score in adherence to the antihypertensive treatments (Table 4). A linear regression established that the number of antihypertensive drugs could statistically significantly predict overall score obtained from the questionnaire, F (1, 251) = 22.29, *P* < 0.018. An extra antihypertensive drug prescription leads to a 0.607 (95% CI: -1.23 to 2.45) point increase in the Hill-Bone questionnaire.

**Table 4:** Summary of simple linear regression analysis

	**Unstandardized Regression Coefficients**		**Standardized Coefficients**	***P ***
	**B**	**SE**	**Beta**	
Age	-0.003	0.055	-0.004	0.952
Gender	0.803	1.164	0.043	0.491
Marital status	-1.477	1.132	-0.082	0.193
Education level	-0.308	0.371	-0.052	0.408
Disease length	0.012	0.099	0.008	0.902
Number of drugs	0.607	0.938	0.041	0.018
BP self-monitoring	0.371	1.223	0.019	0.762

R: 0.041; R2: 2%; *P < 0.05. Abbreviation: SE: Standard error of the coefficient.

The multiple regression model did not significantly predicted overall score, F (14, 164) = 1.077, *P* =0.832, adjusted R^2^ = 0.006. None of the evaluated variables added statistically significantly to the prediction, *P* >0.05. Regression coefficients and standard errors can be found in Table 5.

**Table 5:** Summary of multiple linear regression analysis

	**Unstandardized Regression Coefficients**		**Standardized Coefficients**	***P***
	**B**	**SE**	**Beta**	
Intercept	37.615	6.234		0
Age	0.006	0.094	0.006	0.952
Gender	1.953	1.438	0.109	0.176
Marital status	-2.543	2.838	-0.076	0.372
Occupational status	1.026	0.88	0.113	0.245
Education level	-0.063	0.614	-0.011	0.919
Spouse's education level	-0.463	0.6	-0.078	0.441
Insurance type	0.248	0.61	0.032	0.685
Household-level	0.571	1.716	0.029	0.74
Income satisfaction	-1.324	2.06	-0.053	0.521
Familial history of HTN	-2	1.6	-0.108	0.213
Disease length	-0.034	0.131	-0.022	0.793
Comorbidity	-0.263	1.572	-0.014	0.867
BP self-monitoring	-0.228	1.638	-0.012	0.889

Abbreviation: SE: Standard error of the coefficient. *P < 0.05

## Discussion

HTN is one of the crucial risk factors for atherosclerosis, heart failure, stroke, and renal failure in many countries.^21^ In 2000, HTN was accountable for 4.4% (64 million DALY) of the burden over public health systems, which was calculated to be 1.7% (25 million DALY) for the year 2010 and estimated to be 1.9% (equivalent to 27 million DALY) in the year 2020.^22^ However, many studies have been conducted on the non-adherence with the antihypertensive treatment regimen in other countries, but complications followed by non-adherence to the antihypertensive treatments reveals need for further studies aiming to determine the pitfalls and reduce the burden of CVD on the health systems.^23^

In the present study, we reviewed and determined the factors related to the nonadherence of therapeutic recommendations, both pharmaceutical and non-pharmacological, in patients with HTN in the Family Medicine Clinic of the Tabriz University of Medical Sciences. The statistical analysis showed that the gender of the patients, marital status, the education level of the patients and their spouses did not affect the patients’ follow-up from the therapeutic instructions provided to the patients. Also, the effect of the socioeconomic and financial condition such as occupation and employment status, economic status (household-level), and satisfaction and dissatisfaction with the amount of income of patients were evaluated. No significant relationship observed between the variables mentioned earlier and the adherence to treatment. Since no reliable and well-established criteria has been introduced for assessment of the socioeconomic position in low- and middle-income countries, we included household-level, occupational status and income satisfaction as indicators for individuals socioeconomic condition, which was in accordance with the suggestion of Howe et al.^20^

However, the patients’ benefit from the various type health insurance services had a significant effect on the scores of patients’ adherence to regular follow-up sessions by the physician. However, the disease criteria such as the duration of HTN incidence, the intervals of referral to the doctor, the familial history of HTN in the first-degree relatives, the development of subsequent complications, in addition to blood pressure monitoring at home were evaluated. No significant effect on the quality of the patients’ adherence to the treatment recommendations observed, but taking two or less than two antihypertensive drugs would improve the patient’s overall adherence with the treatment recommendations, especially in the areas of dietary recommendations, as well as the adherence to the appointment keeping, which was in consistent with the results of the study by Bramley et al.^24^ Since previous studies reported high rate of the therapy discontinuation during one-year period in patients with higher number of drugs, it seems that less drug prescription has less destructive effects on patients’ adherence to the therapy.^25,26^

In our study, the results showed that the highest adherence score was in the appointment keeping and the lowest was associated with medication adherence. However, in a study by Kyngas et al on the factors influencing the quality of the treatment adherence in patients with HTN, the adherence for the dietary recommendations had the lowest rate among patients, however, despite our findings, medication adherence had the highest rate of adherence.^27^ Similarly, researchers in previous study revealed that factors such as non-smoking, asymptomatic HTN, higher education level, and female gender are active factors in improvement of the dietary recommendations adherence by patients, but in our study, gender of the patients and the level of education of the people did not correlate with the acceptance of the treatment. The difference between the results of the two studies may be due to the lack of the adequate advised by the physician on the importance of proper use of drugs and adherence to recommendations by the patients and their relatives. Our results proved that higher number of anti-hypertensive drugs increases patients willing to attend regular appointments with their physicians and their adherence to diet suggestions, that leads to increased points in the Hill-Bone questionnaire.

In another study, the role of mental health and the patients’ marital status correlation with adherence to antihypertensive treatments evaluated, and the results indicated that the rate of adherence was higher in married patients.^14^ However, the results of our study showed that not only the marital status of patients does not affect the treatment of patients, but also the level of education of spouses of patients is not in correlation with adherence with the therapeutic guidelines. The reason for this difference between the studies can be directed to the cultural and social differences in our country that affects the relationship between the spouses and the importance of the spouses’ disease to their partners.

In a study by Jokisalo et al evaluating the various causes of non-adherence with antihypertensive therapy in patients including the health system related factors, the results of the study showed a significant relationship between the long-term antihypertensive therapy induced anxiety, the economic problems and decreased rate of patients’ adherence to the treatment.^28^ However, although health system-related factors were not studied in our study, contrary to the previous study, the economic status and income level satisfaction did not affect the acceptance of treatment recommendations from patients. However, we believe that the majority of the patients refer to the family medicine clinics to benefit from the low-cost services of these centers due to lack of financial ability. This fact led to an insignificant effect of the patients’ economic status on treatment adherence in HTN patients, which can be considered as a bias factor and is subjected to the limitations of our study.

Svensson et al conducted a study on the causes associated with adherence with antihypertensive therapy in patients. They obtained using interviewing and counseling tools to identify the patients’ behaviors, and purposes provide better justification for the patients to improve adherence with the treatment of chronic illnesses.^29^ However, in our study, the highest rate of adherence was the appointment keeping of the patients. On this basis, we hypothesized that regular referral of the patients to their physicians provides a great opportunity for justifying patients and improving the overall score of the patients. Similarly, in a systematic review, Rabbia et al emphasized the importance of the physicians’ recommendations to enhance the adherence to the treatment by the patients.^30^

Our study was of some limitations. First, it can be noted that the study was conducted only in university clinics, which is mainly visited by low-income patients, due to the lower expenses which have led to bias in the determination of the economic status effect of treatment adherence. Second, proper and complete instructions from the prescribing physician are the vital factor that accelerates the acceptance of treatment by the patients, which has been not taken into account in our study.

## Conclusion

The medication instructions were the most compliant treatment recommendations among hypertensive patents, however, number of administered drugs significantly affected patients’ adherence to dietary restrictions and appointment keeping.

## Ethical approval

The study was approved by the ethics committee of the Vice Chancellor of Research and Development, Tabriz University of Medical Sciences (IR.TBZMED.REC.1397.201).

## Competing interests

All authors declare no competing financial interests exist.
